# Inspection of Cattle and Sheep for Export

**Published:** 1891-01

**Authors:** 


### EDITORIAL DEPARTMENT.

### INSPECTION OF CATTLE AND SHEEP FOR EXPORT.

The eleventh hour is better than no hour at all, and at last the United States Government has done something to meet the complaints of foreign nations in regard to our food animals, and the restrictions which they have placed against them.

By authority of Section 10 of the Act of Congress approved August 30th, 1890, entitled "An Act providing for the inspection of meats for exportation, prohibiting the importation of adulterated articles of food or drink," etc., Mr. Rusk, the Secretary of Agriculture, has directed the Chief of the Bureau of Animal Industry to cause careful veterinary inspection to be made of all neat cattle and sheep to be exported from the United States to Great Britain, Ireland and the Continent of Europe.

The order and regulations provide for inspection and tagging of the animals, the cleanliness of all means of transport, the investigation of the origin of all cattle shipped abroad, and forbids the clearance of vessels with cattle, unless the latter are accompanied by the proper certificates of the Veterinary Inspector.

Dr. Charles B. Michener has been appointed Veterinary Inspector, Port of New York, and is conveniently established at 18 Broadway, New York. Dr. Michener has been granted rather more liberal power and facilities than we veterinarians are accustomed to expect. He is assisted by Dr. Henry Brister and Dr. Thomas H. Ash. Dr. R. W. Hickman has charge of the inspections at Chicago, where he has established two "shutes" in the stock yards for handling the cattle, and has a force of some eleven lay assistants. They have been obliged to handle over 1,700 cattle a day. The other inspectors are: Dr. Claris, Buffalo; Dr. Armstrong, Pittsburg; Dr. W. B. E. Miller, Philadelphia; Dr. Faville, Baltimore; Dr. Wm. Rose, Kansas City, and Dr. A. H. Rose, Boston.

At inland stock yards the cattle are inspected and tagged before shipment to the seaboard. At ports of export they are again inspected, the tags verified, and a certificate stating that the animals "have passed a careful veterinary examination, and so far as can be ascertained, they have not been exposed to any contagious disease," is furnished the vessel master before clearing. Dr. Wray in London is furnished with duplicate papers, and follows the disposition of the animals in Europe. While the regulations, so far, only apply to cattle and sheep, the act provides for the inspection of hogs and pork in such a liberal manner that we need not apprehend any future trouble for this much abused and maligned animal.

The step which this law, and these regulations make, toward a general inspection of our animal food, opens the doors to a good and valuable livelihood for a large number of young veterinarians, and must indirectly be of considerable importance and value to our veterinary schools, as well as to the welfare of the whole people.

